# Methotrexate Combined with 4-Hydroperoxycyclophosphamide Downregulates Multidrug-Resistance P-Glycoprotein Expression Induced by Methotrexate in Rheumatoid Arthritis Fibroblast-Like Synoviocytes via the JAK2/STAT3 Pathway

**DOI:** 10.1155/2018/3619320

**Published:** 2018-02-18

**Authors:** Kaili Qin, Kailin Chen, Wenpeng Zhao, Xiangcong Zhao, Jing Luo, Qun Wang, Chong Gao, Xiaofeng Li, Caihong Wang

**Affiliations:** ^1^Department of Rheumatology, The Second Hospital of Shanxi Medical University, Taiyuan, Shanxi, China; ^2^Pathology, Joint Program in Transfusion Medicine, Brigham and Women's Hospital/Children's Hospital, Harvard Medical School, Boston, MA, USA

## Abstract

**Objective:**

Rheumatoid arthritis (RA) multidrug resistance is associated with P-glycoprotein (P-gp) overexpression. We investigated the effects of methotrexate (MTX) alone and combined with 4-hydroperoxycyclophosphamide (4-HC) on P-gp expression in fibroblast-like synoviocytes (FLSs) from patients with RA and examined the signaling pathway involved.

**Methods:**

RA-FLSs were treated with MTX, MTX + 4-HC, AG490 + MTX, or AG490 + MTX + 4-HC for 72 h. Proliferation inhibition rates were determined by MTT assay; P-gp expression was measured by flow cytometry and real-time polymerase chain reaction (RT-PCR); JAK2 and STAT3 were measured by RT-PCR and cell-based ELISA to assess STAT3 signaling.

**Results:**

MTX alone significantly induced P-gp expression and mRNA production in RA-FLSs. P-gp expression and mRNA levels were lower in the MTX + 4-HC group than in the MTX-alone group. In contrast to MTX, MTX + 4-HC reduced the STAT3 phosphorylation and downregulated JAK2 and STAT3 mRNA production. Inhibition of constitutively active STAT3 accompanied by 4-HC suppressed P-gp levels in RA-FLSs. The MTT assays revealed no significant differences in proliferation inhibition rates among groups.

**Conclusions:**

The increased anti-P-gp effect of MTX + 4-HC versus MTX alone in RA-FLSs was mediated via inhibition of the JAK2/STAT3 pathway and may have helped reverse MDR in refractory RA patients with high-P-gp levels.

## 1. Introduction

Rheumatoid arthritis (RA) is an autoimmune disease characterized by erosive arthritis. The major pathological feature of RA is chronic inflammation of the synovial tissue and the formation of pannus with erosion of the articular cartilage and bones, ultimately causing joint deformity and dysfunction [1]. The global incidence of RA is 0.5% to 1%, whereas the incidence in China is 0.2% to 0.4%, with an estimated 5 million sufferers [2]. Improvement in the diagnosis and treatment of RA has been the focus of much recent attention.

Early treatment with disease-modifying antirheumatic drugs (DMARDs), such as methotrexate (MTX), can effectively delay disease progression and improve prognosis, and the treatment has attracted an international consensus [3]. However, clinical observations suggest that some patients have a poor or no response to DMARDs, described as multidrug resistance (MDR), which results in refractory RA (RRA) [4]. The mechanisms underlying MDR are complex; however, ATP binding cassette (ABC) transmembrane proteins, particularly the multidrug resistance 1 gene (MDR1), which encodes transmembrane protein P-glycoprotein (P-gp), play an important role [5]. P-gp is a drug efflux pump responsible for the removal of drugs from cells against a concentration gradient; overexpression of P-gp results in low intracellular drug concentrations, leading to drug resistance [5].

Moreover, P-gp regulation is complex. Factors, such as drugs, cytokines, gene polymorphisms, and oncogenes, can affect the expression of P-gp [6]. In addition to serving as the primary treatment for RA, MTX is a specific substrate of P-gp [7]. Several studies have demonstrated P-gp overexpression in the lymphocytes or synovial cells of MTX-resistant RA patients [8–10]. Treatment with the P-gp inhibitors, cyclosporine A and tacrolimus, has been shown to significantly improve the clinical symptoms and laboratory indicators in RRA patients [11, 12]. These findings suggest that P-gp is involved in MTX resistance in RA. However, the mechanisms by which MTX induces P-gp activation remain unclear. As a dihydrofolate reductase inhibitor, MTX inhibits the synthesis of DNA, thus exerting pharmacological effects; however, previous studies have suggested a possible connection between MTX and the Janus kinase 2-signal transducer and activator of the transcription 3 **(**JAK2/STAT3) pathway, although there is controversy regarding whether MTX play an activating [13] or inhibitory [14, 15] role. Despite contradictory evidence, these findings provide new insights into the pharmacology of MTX. Furthermore, the JAK/STAT pathway is closely associated with P-gp production and drug resistance [16–21]. Given these findings, we hypothesized that MTX activates P-gp production via the JAK2/STAT3 pathway.

High-dose cyclophosphamide (CTX) (1~2 g/d) is a DMARD generally reserved for severe RA cases in spite of its side effects. For the past 10 years, the Department of Rheumatism at our hospital has used MTX and low-dose CTX (0.2~0.4 g) combination therapy administered periodically (3 weeks). The efficacy and safety of this therapy have been widely verified [22–24]. Importantly, this therapy significantly reduces the expression of P-gp in RRA [22–24], although the underlying mechanisms are not well understood. We compared the effects of MTX alone and MTX plus 4-hydroperoxycyclophosphamide (4-HC), an active metabolite of CTX *in vitro*, on the expression of P-gp and investigated the involvement of the JAK2/STAT3 pathway to clarify the mechanisms underlying the MXT + 4-HC-induced modulation of P-gp expression in RA fibroblast-like synoviocytes (FLSs).

## 2. Materials and Methods

### 2.1. Human Tissue Collection and Ethics Statement

Synovial tissue specimens were obtained by fine-needle aspiration biopsy (FNAB) from three patients with recent-onset arthritis who were naive to DMARDs, corticosteroids, and biological agents. The patients were recruited between July 2016 and December 2016 from the Department of Rheumatology at the Second Hospital of Shanxi Medical University. RA was diagnosed according to the 1987 American College of Rheumatology classification criteria [25]. The patients provided informed consent for the use of their tissue, and the study was reviewed and approved by University Institutional Review Board.

### 2.2. Reagents and Antibodies

MTX was purchased from Sigma-Aldrich (St. Louis, MO, USA). 4-HC was purchased from Carbosynth (Compton, Berkshire, UK). AG490 (a JAK2/STAT3 pathway inhibitor) was purchased from R&D Systems Inc. (Minneapolis, MN, USA). Antibodies against multidrug-resistance-associated protein 1 (MRP1) (anti-MDR1 [U1C2]) were purchased from Santa Cruz Biotechnology (Santa Cruz, CA, USA). 3-(4,5-Dimethylthiazol-2-yl)-2,5-diphenyltetrazolium bromide (MTT) was purchased from Solarbio Science and Technology Co. Ltd. (Beijing, China). A cell-based enzyme-linked immunosorbent assay (ELISA) kit was purchased from RayBiotech (Norcross, GA, USA).

### 2.3. Cell Culture and Treatment

FLSs were isolated from synovial tissue specimens obtained from RA patients. Cells were cultured *in vitro* and used at passages 5-6 in the experiments. The drug concentrations used in the experiments were MTX (0.01 *μ*g/mL), 4-HC (1 *μ*g/mL), and AG490 (50 *μ*M). RA-FLSs were randomly divided into five treatment groups: control cells (group A); cells cultured with MTX alone (group B); cells cocultured with MTX + 4-HC (group C); cells pretreated with the JAK2/STAT3 signaling pathway inhibitor, AG490, for 30 min before adding MTX alone (group D); or cells pretreated with the JAK2/STAT3 signaling pathway inhibitor, AG490, for 30 min before adding MTX + 4-HC (group E). FLSs were incubated at 37°C in 5% CO_2_-saturated humidity for 72 h before harvest. The effect of MTX ± 4-HC on P-gp and the JAK2/STAT3 pathway were observed. Cells were then collected, and (1) FLS cellular proliferation inhibition rates were assessed by MTT assay; (2) P-gp expression was measured by flow cytometry; (3) mRNA production of P-gp, JAK2, and STAT3 was measured by real-time polymerase chain reaction (RT-PCR); and (4) the protein content of phosphorylated STAT3 (p-STAT3) in FLSs was determined by cell-based ELISA.

### 2.4. Cell Viability Assay

Cell viability was measured by MTT assay. Cells were seeded at 5 × 10^4^ cells/well in 96-well plates, incubated overnight, and then exposed to the indicated concentrations of MTX and 4-HC for 72 h. Thereafter, 20 *μ*L of MTT solution (5 mg/mL) was added to each well and incubated for another 4 h at 37°C. After removal of the culture medium, cells were lysed in 200 *μ*L of dimethyl sulfoxide, and the optical density (OD) was measured at 570 nm using a microplate reader (Thermo Fisher Scientific, Waltham, MA, USA). The following formula was used: cellular proliferation inhibition rates = (OD of the control group − OD of the experimental sample)/OD of the control group × 100%.

### 2.5. Flow Cytometry

After being cultured for 72 h, cells were collected and washed with excess phosphate-buffered saline (PBS) twice and then incubated with 20 *μ*L of phycoerythrin- (PE-) conjugated human anti-P-gp mAb MDR-1 (UIC2) and 20 *μ*L of PE-conjugated-matched isotype control antibody (normal mouse IgG2a) for 30 min at room temperature. Then, the cells were washed twice in PBS and subsequently fixed with PBS and analyzed on a FACSCalibur cytometer (Becton Dickinson, Franklin Lakes, NJ, USA). Cell Quest Pro software (BD Biosciences, San Jose, CA, USA) was used for data acquisition and analysis. At least 10,000 cells were collected for analysis and separated according to their forward and side scatter characteristics. The data are expressed as the percentage of positive cells and the relative fluorescence intensity (RFI).

### 2.6. Relative Quantitative Real-Time PCR

Total RNA was isolated from the RA-FLSs using TRIzol reagent (Takara, Kyoto, Japan). The complementary DNA was synthesized and amplified by real-time PCR using a Prime Script RT Reagent Kit (Takara). Real-time PCR reactions were performed in a CFX96 real-time PCR detection system (Bio-Rad, Hercules, CA, USA) using SYBR Premix Ex Taq II (Takara). The real-time-PCR conditions included 95°C for 10 min, followed by 40 cycles of 95°C for 15 s and 60°C for 31 s. Primer specificity was monitored using product-melting curves in each reaction well. Raw data were normalized and expressed relative to the housekeeping gene *β*-actin as two ^−ΔΔ*C*t^ values. The relative amplification efficiencies of the primers were tested and shown to be similar.

The following primers were used:

1. Human-MDR1-F 5′AGTTGAGTGGTGGGCAGAAG 3′; human-MDR1-R 5′ACCACTGCTTCGCTTTCTGT 3′
2. Human-*β*-actin-F 5′AGCGAGCATCCCCCAAAGTT 3′; human-*β*-actin-R 5′GGGCACGAAGGCTCATCATT 3′
3. Human-JAK2-F 5′AGCCTATCGGCATGGAATATCT 3′; human-JAK2-R 5′TAACACTGCCATCCCAAGACA 3′
4. Human-STAT3-F 5′CTTTGAGACCGAGGTGTATCACC 3′; human-STAT3-R 5′GGTCAGCATGTTGTACCACAGG 3′

### 2.7. Cell-Based ELISA

Total p-STAT3 levels in whole cells were measured by ELISA-based assay using fluorogenic substrates according to the manufacturer's protocol (RayBiotech). Cells were grown in microplates under the various conditions and fixed, quenched, and blocked; they were next incubated simultaneously with, first, p-STAT3 primary antibodies and, then, secondary antibodies. After the addition of the 3,3′,5,5′-tetramethylbenzidine substrate, fluorescence was measured using a microplate reader (Thermo Fisher Scientific) at 450 nm, and p-STAT3 fluorescence was normalized after background subtraction.

### 2.8. Statistical Analysis

Means (±standard deviations (SD)) were calculated. The statistical analyses were performed using the Statistical Package for the Social Sciences version 17.0 (SPSS, Chicago, IL, USA). The Kolmogorov–Smirnov test was used to determine the normality of the distribution of the data, and Levene's *t*-test was used to test the homogeneity of variance. One-way analyses of variance (ANOVAs) were used for between-group comparisons, and the least significant difference (LSD) *t*-test was used for within-group comparisons. *P* values < 0.05 were considered to indicate statistical significance.

## 3. Results

### 3.1. MTX and MTX + 4-HC Inhibited Cell Viability

**Figure 1:**
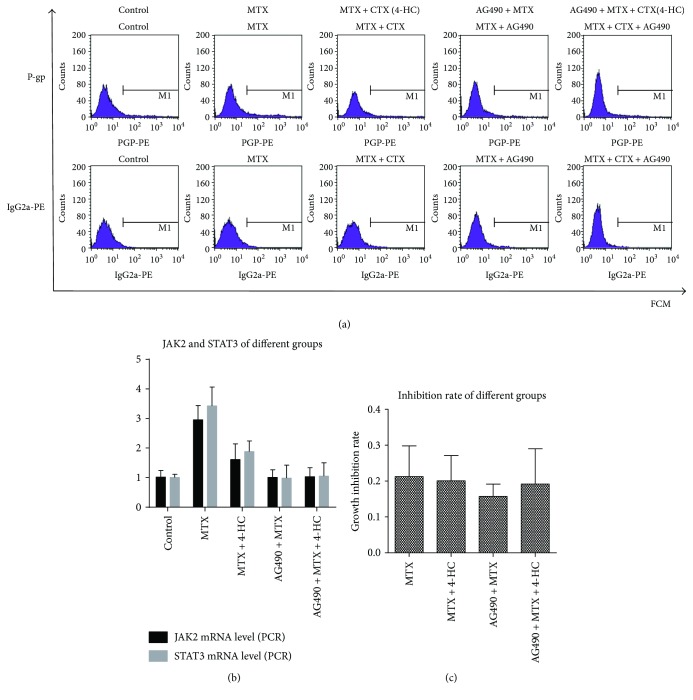
Effects of methotrexate (MTX) (a) and/or 4-hydroperoxycyclophosphamide (4-HC) (b) on P-glycoprotein (P-gp) levels and cell viability(c) of rheumatoid arthritis fibroblast-like synovial cells (RA-FLSs). Compared with the cell control group, the other medicine groups showed higher levels of P-gp expression and P-gp mRNA-production; the differences were statistically significant (*P* < 0.05); compared with MTX group, the MTX + 4-HC group showed lower levels of P-gp expression and P-gp mRNA-production; the differences were statistically significant (*P* < 0.05); after adding pathway inhibitors, P-gp expression and P-gp mRNA-production were lower than the MTX ± 4-HC group, the differences were statistically significant (*P* < 0.05); comparison between groups showed no differences in cellular inhibitory rates (*P* > 0.05).

RA-FLSs were treated with MTX, MTX + 4-HC, AG490 + MTX, or AG490 + MTX + 4-HC for 72 h. MTT assays for cell viability revealed that both MTX alone and in combination with 4-HC inhibited cell growth in the presence and absence of AG490 (Figure 1(c)). The cellular inhibition rates for MTX-treated (0.21 ± 0.09) and MTX + 4-HC-treated (0.20 ± 0.07) cells were not significantly different.

### 3.2. MTX Promoted the Level of P-gp More than MTX + 4-HC

P-gp expression was determined by flow cytometry (Figures 1(a) and 1(b)), and mRNA-production levels were measured by RT-PCR (Figure 1(b)). The RFI values revealed that, compared with that in the cell control group (1.88 ± 0.47), P-gp expression was significantly higher in the MTX (4.94 ± 0.19) and MTX + 4-HC (3.06 ± 0.34) groups (*P* < 0.05). Similarly, P-gp mRNA production was higher in the MTX (3.31 ± 0.53) and the MTX + 4-HC (2.00 ± 0.53) groups compared with the cell control group (1.03 ± 0.28; *P* < 0.05). Additionally, the analyses revealed that the P-gp expression and P-gp mRNA production levels were lower in the MTX + 4-HC group than in the MTX group (*P* < 0.05). After pretreatment with the pathway inhibitor, P-gp expression and P-gp mRNA-production were significantly lower in the MTX ± 4-HC group (*P* < 0.05): The RFIs measured by flow cytometry finding were as follows: MTX (4.94 ± 0.19) versus AG490 + MTX (1.9 ± 0.33) and MTX + 4-HC (3.06 ± 0.34) versus AG490 + MTX + 4-HC (2.23 ± 0.42); mRNA production showed that MTX (3.31 ± 0.53) versus AG490 + MTX (3.13 ± 0.32) and MTX + 4-HC (2.00 ± 0.53) versus AG490 + MTX + 4-HC (1.14 ± 0.24), indicating that inhibition of the JAK2/STAT3 pathway was related to P-gp levels.

### 3.3. JAK2/STAT3 Pathway Involvement in MTX- and MTX + 4-HC-Induced Induction of P-gp

**Figure 2:**
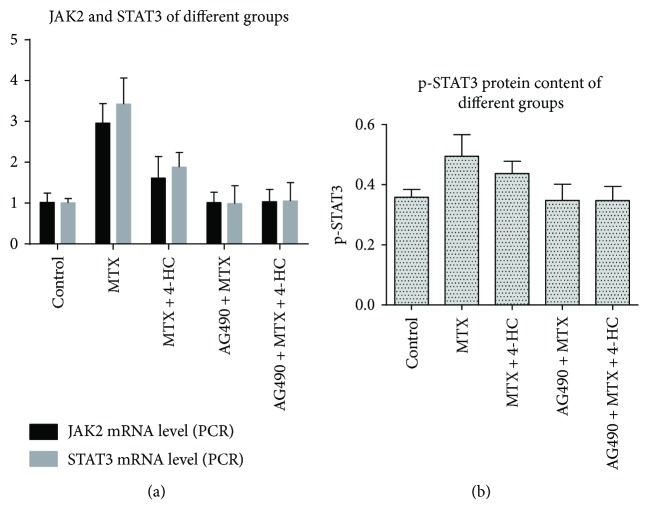
Effects of methotrexate (MTX) and/or 4-hydroperoxycyclophosphamide (4-HC) on JAK2/STAT3 mRNA-production (a) and p-STAT3 protein content (b) of rheumatoid arthritis fibroblast-like synovial cells (RA-FLSs). Compared with the cell control group, the MTX ± 4-HC group showed higher levels of JAK2/STAT3 mRNA-production and p-STAT3 protein content; the differences were statistically significant (*P* < 0.05); compared with the MTX group, the MTX + 4-HC group showed lower levels of JAK2/STAT3 mRNA-production and p-STAT3 protein content; the differences were statistically significant (*P* < 0.05); after adding pathway inhibitors, JAK2/STAT3 mRNA-production and p-STAT3 protein content were lower than those of the MTX ± 4-HC group; the differences were statistically significant (*P* < 0.05).

The JAK2/STAT3 pathway inhibitor, AG490, has been shown to influence P-gp expression levels in the above experiments; thus, we further investigated the effect of AG490 on MTX and MTX + 4-HC activity in RA-FLSs (Figure 2(a)). After pretreatment with pathway inhibitors, JAK2 and STAT3 mRNA production was significantly lower in the MTX and MTX + 4-HC groups (*P* < 0.05): for JAK2, MTX (2.95 ± 0.48) versus AG490 + MTX (1.01 ± 0.26) and MTX + 4-HC (1.60 ± 0.53) versus AG490 + MTX + 4-HC (1.03 ± 0.30); for STAT3, MTX (3.43 ± 0.64) versus AG490 + MTX (0.99 ± 0.44) and MTX + 4-HC (1.90 ± 0.36) versus AG490 + MTX + 4-HC (1.05 ± 0.45). Moreover, the assessment of activated STAT3 (pSTAT3) by cell-based ELISA revealed similar results (Figure 2(b)): MTX (0.49 ± 0.07) versus AG490 + MTX (0.35 ± 0.05) and MTX + 4-HC (0.44 ± 0.04) versus AG490 + MTX + 4-HC (0.35 ± 0.05). These findings suggest that the JAK2/STAT3 pathway was involved in the MTX- and MTX + 4-HC-induced induction of P-gp.

## 4. Discussion

RA is characterized by synovitis with multiple system involvement [1]. MTX, CTX, and other DMARDs have been used to effectively treat the clinical symptoms of RA for decades. However, MDR to DMARDs poses a significant clinical challenge. From the pharmacodynamic perspective, MDR includes drug transport *in vivo*, drug intake and efflux by target cells, changes in drug activity, and other abnormal aspects [26]. The ABC transmembrane transporter superfamily has been shown to increase intracellular drug efflux and decrease intracellular drug concentration [27]. This superfamily contains more than 100 types of membrane transporters or channels, which are divided into seven subfamilies according to sequence similarity (ABC A–G). Of these, ABCB1/P-gp, ABCC1/MRP1, and ABCG2/breast cancer resistance protein are primarily associated with MDR. The role of the ABC transmembrane transporter superfamily has been widely studied in neoplastic diseases, infection, and inflammatory diseases [27]; however, its impact on RA and other autoimmune diseases has been investigated only recently. We investigated the mechanisms underlying P-gp expression and regulation in RA-FLSs with the goal of providing new insights into the treatment of RA and of RRA, in particular.

P-gp is composed of 1280 amino acids and is one of the most widely studied transporters encoded by the MDR1 gene on the long arm of chromosome 7(7q21). The glycoprotein is 170 kD and contains 12 transmembrane regions, two cell plasma nucleotide-binding domains, and two homology dimers, all of which constitute the channel for substrate transport across membrane. P-gp is expressed in the blood–brain barrier, gastrointestinal tract, kidney, liver, pancreas, and cancer cells [28]. However, recent studies have shown that P-gp-induced MDR is not limited to intracellular transport. P-gp located on lysosomes can transport intracellular drugs into the lysosomes, where they can accumulate and are metabolized, inhibiting their pharmacological effects, or can act to increase lysosomal membrane permeability, causing cell death [29]. Furthermore, cellular proliferation affects drug resistance, reducing cellular apoptosis, or speeding cellular proliferation activity could enhance drug resistance [30]. Therefore, MTT assays were used to assess the inhibitory actions of MTX ± 4-HC in RA-FLSs *in vitro* to control for the effect of cell proliferation. Our finding that the effect of MTX + 4-HC on FLS proliferation was not influenced by the JAK2/STAT3 pathway inhibitor, AG490, allowed us to more objectively investigate the effect of drugs on P-gp activity.

Several drug substrates affect P-gp activity, including anticancer drugs, antibiotics, and a variety of DMARDs. The effect of the substrate depends on the P-gp protein binding site. For example, compounds that bind to the main binding cavity (MBC) at the top of P-gp are strong substrates, whereas nonsubstrates tend to bind to middle sites, and half substrates bind at both sites. P-gp reacts differently to the three types of substrate [31]. Controversy remains as to whether MTX is a complete substrate for P-gp, with some studies suggesting it is [7, 28], and others arguing that it is not [31]; nevertheless, it is clear that MTX has an obvious effect on P-gp [8–10]. The interaction between MTX and P-gp may involve factors in addition to the efflux pump. A recent study of tumor cells established a drug-resistant cell-line culture that overexpressed P-gp *in vitro*, and the cells were pretreated with noncytotoxic concentrations or low doses of active metabolites of CTX (i.e., 4-HC). The study found that the treatment improved the effect of etoposide. The authors speculated that the effect might be related to 4-HC-induced inhibition of the cell cycle [32].

In fact, both MTX and CTX are immunosuppressive agents and are often administered at high doses to kill tumor cells in the treatment of tumors and hematological diseases [33, 34]. At low doses, MTX and CTX have broad therapeutic roles in autoimmune diseases. Based on the principles of cell proliferation kinetics, as a cell cycle-specific drug, MTX acts primarily at the G1 and S phases, whereas CTX is a cell cycle-nonspecific drug that kills G0- and proliferation-phase cells but has a stronger killing effect on S-phase cells. Therefore, in theory, MTX (10~15 mg/week) arrests the majority of cells in the G1 and S phases, and the low dose of CTX (0.2~0.4 g/3 weeks) then effectively kills the cells arrested by MTX in the S phase, improving the curative effect of the drugs. Furthermore, MTX and CTX have different pharmacological mechanisms and nonoverlapping side effects; thus, low and interval doses allow normal tissue and organs to recover. As such, the therapy is effective with fewer adverse reactions [22–24]. These findings provide the theoretical basis for the MTX/CTX combination therapy used in our hospital department. Our findings provide insight into the mechanisms underlying P-gp regulation by MTX/CTX and reduced drug resistance. We found that, although MTX alone tended to induce P-gp overexpression in RA-FLSs, MTX + CTX could reduce P-gp overexpression than the MTX monotherapy, providing support for the efficacy of the combined treatment.

The JAK2/STAT3 pathway is essential for cellular biological activities, including cellular growth and metabolism. Recent studies have revealed a connection between MTX and the JAK2/STAT3 pathway, although the role of MTX is controversial, with some evidence suggesting that MTX activates the STAT3 pathway [13] and other studies finding that the drug inhibits the pathway [14, 15]. Moreover, several recent studies have confirmed the involvement of the JAK/STAT pathway in P-gp production and MDR [16–21]. In light of these findings, we hypothesized that MTX induces P-gp production via the JAK2/STAT3 pathway. To test this, we pretreated samples with the JAK2/STAT3 inhibitor, AG490, and then added MTX ± 4-HC to the cells; we found that the P-gp expression and mRNA production decreased. At the same time, JAK2 and STAT3 mRNA and p-STAT3 protein levels decreased, indicating that the JAK2/STAT3 pathway was involved in MTX ± 4-HC-induced P-gp expression. Furthermore, the presence of 4-HC decreased the MTX-induced increase in P-gp and the activation of the JAK2/STAT3 pathway. What is noteworthy is that the JAK2/STAT3 pathway plays an important role in the pathogenesis of RA; thus, specific pathway inhibitors are potential treatments for RA. Some JAK inhibitors have been used in early clinical trials and phase-three trials [35, 36]. We believe that the JAK/STAT pathway and its related enzymes may provide novel targets for the treatment of RA; however, further basic research is needed before clinical applications can be developed.

## 5. Conclusions

MTX alone enhanced P-gp expression in synocytes in patients with RA, and the JAK2/STAT3 pathway plays a vital role in this process. MTX + 4-HC had a greater anti-P-gp effect than MTX; this effect was mediated via inhibition of the JAK2/STAT3 pathway and has potential therapeutic value for the reversal of MDR in RA, particularly in RRA patients with high P-gp levels.

## Additional Points

*Provenance and Peer Review*. The paper is not commissioned or externally peer reviewed. *Data Sharing Statement*. No additional data are available.
